# Sphingosine-1-Phosphate Transporters as Targets for Cancer Therapy

**DOI:** 10.1155/2014/651727

**Published:** 2014-07-15

**Authors:** Masayuki Nagahashi, Kazuaki Takabe, Krista P. Terracina, Daiki Soma, Yuki Hirose, Takashi Kobayashi, Yasunobu Matsuda, Toshifumi Wakai

**Affiliations:** ^1^Division of Digestive and General Surgery, Niigata University Graduate School of Medical and Dental Sciences, 1-757 Asahimachi-dori, Chuo-Ku, Niigata 951-8510, Japan; ^2^Division of Surgical Oncology, Department of Surgery, Virginia Commonwealth University School of Medicine and the Massey Cancer Center, 1200 E. Broad Street, Richmond, VA 23219, USA; ^3^Department of Medical Technology, Niigata University Graduate School of Health Sciences, 2-746 Asahimachi-dori, Chuo-Ku, Niigata 951-8518, Japan

## Abstract

Sphingosine-1-phosphate (S1P) is a pleiotropic lipid mediator that regulates cell survival, migration, the recruitment of immune cells, angiogenesis, and lymphangiogenesis, all of which are involved in cancer progression. S1P is generated inside cancer cells by sphingosine kinases then exported outside of the cell into the tumor microenvironment where it binds to any of five G protein coupled receptors and proceeds to regulate a variety of functions. We have recently reported on the mechanisms underlying the “inside-out” signaling of S1P, its export through the plasma membrane, and its interaction with cell surface receptors. Membrane lipids, including S1P, do not spontaneously exchange through lipid bilayers since the polar head groups do not readily go through the hydrophobic interior of the plasma membrane. Instead, specific transporter proteins exist on the membrane to exchange these lipids. This review summarizes what is known regarding S1P transport through the cell membrane via ATP-binding cassette transporters and the spinster 2 transporter and discusses the roles for these transporters in cancer and in the tumor microenvironment. Based on our research and the emerging understanding of the role of S1P signaling in cancer and in the tumor microenvironment, S1P transporters and S1P signaling hold promise as new therapeutic targets for cancer drug development.

## 1. Introduction

It is well recognized that the tumor microenvironment (TME) plays a key role in cancer progression and metastasis [1–3]. Tumors influence the surrounding microenvironment through the release of extracellular signals, such as cytokines, chemokines, and lipid mediators [4–7]. These bioactive molecules secreted from cancer cells and noncancer components in the TME, such as blood vessels, lymphatic vessels, and inflammatory cells, are considered to be potential therapeutic targets.

**Figure 1:**
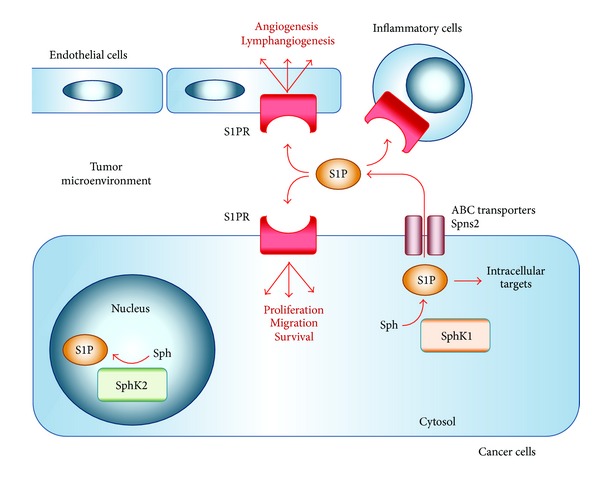
“Inside-out” signaling of sphingosine-1-phosphate (S1P). S1P is generated from Sph (sphingosine) by SphK1 (sphingosine kinase 1) in the cytosol of cancer cells and exported via ABC (ATP-binding cassette) transporters or Spns2 (spinster 2) outside of cells (tumor microenvironment). S1P stimulates specific S1P receptors (S1PR1-5) to promote numerous cellular functions, such as cell proliferation, migration, angiogenesis, and lymphangiogenesis.

Sphingosine-1-phosphate (S1P) has emerged as a new player in the TME and cancer progression during the last decade [4]. S1P generated inside cells is exported outside of cells into the TME where it stimulates specific S1P receptors on the cell surface (Figure 1). This “inside-out” signaling of S1P is considered to play a fundamental role in cancer progression [8]. To date, several S1P transporters have been identified [9–13]. Here, we review the “inside-out” signaling of S1P with a focus on S1P transporters. We will discuss the importance of S1P signaling in cancer and the possibility of targeting S1P transporters for cancer treatment.

## 2. “Inside-Out” Signaling of S1P

**Figure 2:**
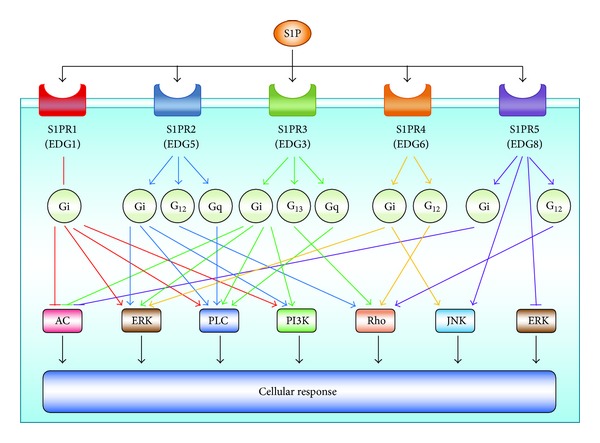
Downstream signaling pathways of sphingosine-1-phosphate (S1P) receptors. S1P is a ligand for the five different specific G protein coupled receptors: S1PR1/EDG1; S1PR2/EDG5; S1PR3/EDG3; S1PR4/EDG6; and S1PR5/EDG8. Each S1P receptor is coupled to different G proteins, which regulate activation or inhibition of the indicated downstream signaling pathways, such as adenylyl cyclase-cyclic AMP, AC; extracellular signal-regulated kinase, ERK; phospholipase C, PLC; phosphatidylinositol 3-kinase, PI3K; the small GTPases of the Rho family; and Jun amino terminal kinase, JNK. Only a few examples of these pathways are illustrated.

S1P is a pleiotropic lipid mediator that regulates cell survival, proliferation, migration, angiogenesis, lymphangiogenesis, and the recruitment of immune cells (Figure 1), making it involved in many physiological and pathological conditions including immune function, inflammation, and cancer [14–19]. S1P is generated from sphingosine inside the cells by two sphingosine kinases (SphK1 and SphK2) [20–23]. The balance between the levels of S1P and its metabolic precursors of ceramide and sphingosine has been regarded as a rheostat that could determine whether a cell proliferates or dies [24]. S1P generated within cells is exported out from cells via S1P transporters on the cell membrane, which will be described in detail later in this review. S1P outside of the cells can stimulate any of five specific G protein coupled receptors (S1PR1-5) [10, 12], with each S1P receptor coupled to different G proteins, which regulate activation or inhibition of the downstream intracellular signaling involved in various cellular functions (Figure 2).

Cancer cells and each type of cell in the TME, such as inflammatory cells and endothelial cells, express different combinations of S1P receptors, which contribute to each cellular function regulated by S1P. For example, S1PR1 is important for B and T lymphocyte egression from secondary lymphatic organs, such as lymph nodes [25]. In endothelial cells, S1PR1 and S1PR2 are known to play an important role in vascular development [26–29]. Stimulation of S1PR1 and/or S1PR3 often promotes cell proliferation and migration in normal and cancer cells, while S1PR2 may inhibit the signaling that promotes cell proliferation and migration [30–32]. Altogether, this “inside-out” signaling of S1P plays a pivotal role in cancer cells and in the TME by stimulating the S1P receptors on each type of cell [33, 34].

In addition to its “inside-out” signaling, S1P is also known to have a variety of intracellular functions. Intracellular S1P produced by SphK1 can bind to TRAF2 (tumor necrosis factor receptor-associated factor 2) and function as a cofactor required for its E3 ubiquitin ligase activity and consequently, Lys-63-linked polyubiquitination of RIP1 (receptor-interacting protein 1) and NF-*κ*B (nuclear factor kappa-light-chain-enhancer of activated B cells) activation [35]. Similarly, it has been shown that S1P enhances cIAP2 (cellular inhibitor of apoptosis 2) mediated K63-linked polyubiquitination of IRF-1 (interferon regulatory factor-1), which is essential for IL-1-induced production of chemokines CXCL10 and CCL5 [36]. Furthermore, S1P produced by SphK2 in the nucleus acts as an endogenous inhibitor of specific histone deacetylases (HDAC1 and HDAC2), thereby regulating gene transcription, including that of the cyclin dependent kinase inhibitor p21 [37].

The relative levels of S1P within body fluids and tissues are important to a variety of physiologic processes [25, 38]. Levels of S1P are maintained by S1P synthesis and degradation, which create an S1P gradient within the tissue [39]. S1P is dephosphorylated to regenerate sphingosine by S1P phosphatases (SPPs) and/or lipid phosphate phosphatases (LPPs). S1P is also irreversibly degraded to hexadecenal and ethanolamine phosphate by S1P lyase (SPL). The current consensus is that trafficking of immune cells is controlled by this S1P gradient. For example, in the blood and lymph, S1P levels are relatively high, but in secondary lymphatic tissue, such as lymph nodes and the thymus, S1P is maintained at very low levels [40]. Importantly, any inhibition of S1P signaling, and therefore altering of this S1P gradient, results in alteration of immune cell trafficking. S1P also regulates vascular integrity. In the plasma, a decrease in the amount of S1P causes increased vascular permeability, likely due to loss of signaling through S1PR1 on endothelial cells [41–43]. S1P through its tissue gradients, intracellular functions, and “inside-out” signaling is important in both physiologic and pathologic processes.

## 3. Export of S1P via ATP-Binding Cassette (ABC) Transporters and Spinster 2 (Spns2) Transporter

Until recently, the process through which S1P produced inside cells by the two SphKs reaches its receptors on the cell surface remained obscure. Membrane lipids, including S1P, do not spontaneously exchange through the lipid bilayers of the plasma membrane since the polar head groups do not readily go through the hydrophobic interior. Though there are many transporter proteins on the membrane for lipid exchange [9], sphingosine is known to spontaneously translocate without the aid of a transporter across intracellular membranes when added to cells or produced intracellularly [9].

Studies from several laboratories, including ours, have suggested the involvement of ABC transporters in the export of S1P from various types of cells* in vitro* [10]. S1P has been shown to be exported from mast cells via ABCC1 (also known as multidrug resistant protein 1; MRP1) [9], from astrocytes via ABCA1 [44], from endothelial cells via ABCA1 and ABCC1 [45], and from thyroid carcinoma cells via ABCC1 [46]. Using pharmacological and molecular approaches, we demonstrated that ABCC1 and ABCG2 (also known as breast cancer resistance protein; BCRP) are involved in estradiol-mediated transport of S1P and dihydro-S1P out of MCF-7 human breast cancer cells [12]. S1P is exported from erythrocytes and platelets by other transporters in the ABC transporter family [47, 48]. In erythrocytes, S1P is exported by an ATP-dependent and vanadate- and glyburide-sensitive transporter [47], while in platelets, S1P export requires an extracellular stimulus such as thrombin and is exported through two independent transporters, a Ca^2+^-dependent transporter and an ATP-dependent glyburide-sensitive transporter [48, 49]. Collectively, these studies suggest that members of the large family of ABC transporters are responsible for export of S1P in various types of cells; however, in studies using mice with ABC transporter deficiencies, including animals with knockout of ABCA1, ABCA7, and ABCC1, S1P levels and related functions have been found to be unaltered [45], indicating the existence of compensatory mechanisms with other transporters.

Spns2, a member of the MFS (major facilitator superfamily) that does not have a typical ATP binding motif, has been recently discovered to export S1P from cells [11, 50–52]. Spns2 was identified independently by two groups, both of whom revealed that it transports S1P through observations in zebrafish. They showed that a mutation in Spns2 caused abnormal development resulting in cardia bifida (two hearts) [53] and that the phenotype of the Spns2 mutation is rescued by providing exogenous S1P [11]. The cardia bifida phenotype in the Spns2 knockout zebrafish was the clue that linked Spns2 to S1P, since the same phenotype was seen in S1PR2 knockout zebrafish [11, 53]. We have also shown that Spns2 can export endogenous S1P and dihydro-S1P from cells [13]. In addition, human Spns2 can transport several S1P analogues, including phosphorylated FTY720 [54]. Importantly, Spns2 was the first S1P transporter discovered to be physiologically functional* in vivo*, in contrast to the ABC transporters [54].

It has been suggested that Spns2 is important for vascular development [29]. We have observed that lymph nodes from Spns2 knockout mice have aberrant lymphatic sinuses that appear collapsed, with reduced numbers of lymphocytes [13]. Our data suggest that Spns2 is an S1P transporter* in vivo* that plays a role in regulation of levels of S1P not only in the blood, but also in the lymph nodes and lymphatic fluid, influencing lymphocyte trafficking and lymphatic vessel network organization [13]. The recent finding that blood endothelial cells purified from the aorta of Spns2-deficient mice are unable to release S1P [52] seems to support a strong role for Spns2 in S1P regulation. The levels of S1P in the plasma of Spns2 knockout mice have been observed to be decreased to 60% of that of wild type mice with endothelial cells contributing 40% of the total plasma S1P [54]. There was no difference between Spns2 knockout mice and wild type in S1P release activity in both erythrocytes and platelets, showing that a disruption of Spns2 does not affect the S1P release from erythrocytes or platelets [52]. Furthermore, bone marrow reconstitution studies revealed that Spns2 was not involved in S1P release from blood cells and suggested a role for Spns2 in other cells [51]. Consistent with these data, specific deletion of Spns2 on endothelial cells has been shown to result in a lack of lymphocyte egress, mimicking observations in global Spns2-knockout mice. These data suggest that Spns2 functions in endothelial cells, not blood cells, to establish the S1P gradient required for T and B cells to egress from their respective lymphoid organs [51].

## 4. Targeting S1P Transporters for Treatment of Cancer Patients

There have been an increasing number of studies, implicating roles for S1P in different stages of cancer progression in subtypes of both adult and pediatric malignancies. SphKs and S1P signaling have been suggested to also have a role in acquisition of drug resistance [55, 56]. There is a growing body of literature, with several clinical and pathological reports revealing the importance of SphK1 on cancer metastasis and prognosis [57–61]. Previous clinical studies have shown that SphK1 is overexpressed in human breast cancer and its expression correlates with poor patient outcomes [58, 62].

Studies linking the molecular interactions of S1P signaling with other oncogenic pathways, such as Ras, STAT3 (signal transducer and activator of transcription 3), NF-*κ*B, and estrogen signaling, have been published. K-Ras mutations are known to increase the production of S1P in a SphK1-dependent manner and expression of the K-Ras oncogene leads to plasma membrane localization of SphK1. The RAS/RAF (rapidly accelerated fibrosarcoma)/MEK (mitogen-activated protein kinases)/ERK (extracellular signal-regulated kinases) pathway likely mediates this process, as constitutively active B-Raf or MEK are capable of activating SphK1 [63]. S1P produced by upregulated SphK1 in tumor cells activates S1PR1, which has been shown to lead to activation of STAT3 [34]. S1P is also involved in the activation of NF-*κ*B, thereby regulating the transcription of the proinflammatory cytokines TNF-*α* (tumor necrosis factor-alpha) and IL-6 (interleukin-6) [34]. Estradiol is known to also stimulate SphK1 activation and the release of S1P, through which estradiol is capable of activating the S1PR3, resulting in EGFR transactivation in a matrix metalloprotease-dependent manner [31]. Altogether, these molecular interactions of S1P signaling with other oncogenic pathways suggest the importance of S1P signaling in cancer.

Communication among tumor cells, the host microenvironment, and inflammatory cells via systemic S1P regulates metastasis. S1P generated in cancer cells is secreted into tissue interstitial fluid in the body and affects the TME by altering immune cells, evoking inflammation, and inducing angiogenesis and lymphangiogenesis thereby promoting cancer metastasis [34, 64]. We have recently measured S1P levels within tumor interstitial fluid and found that significantly higher levels of S1P compared with the interstitial fluid of normal tissue and that inhibiting the “inside-out” signaling of S1P by FTY720 (known as fingolimod) significantly decreased S1P levels in the tumor interstitial fluid (unpublished data). Altogether, the “inside-out” signaling of S1P with overexpressed SphK1 and S1P transporters plays an important role in cancer progression through its effects on the TME.

It has recently been reported that a specific pharmacological inhibitor of SphK1 had no effect on cell proliferation* in vitro* [65]. This result generated some arguments that S1P, the product of SphK1, may not be an ideal anticancer target. On the other hand, targeting S1P signaling leads to a significant suppression of cancer progression* in vivo* [64], especially under conditions in which cancer is associated with inflammation [34]. Considering the importance of “inside-out” signaling of S1P within the TME through promotion of angiogenesis and lymphangiogenesis [4, 66, 67], the effects of targeting S1P signaling may only be adequately assessed* in vivo*.

ABC transporters were originally described as multidrug resistance genes and have been shown to be overexpressed in various solid and hematological cancers [68]. Expression of ABC transporters has been correlated with resistance to chemotherapies and poor prognosis for patients with certain types of cancer [68]. ABCB1, also known as multidrug resistant gene 1 (MDR1), has been targeted in a number of clinical trials that failed to demonstrate significant benefit [69]. Since it has been suggested that several ABC transporters are involved in secretion of S1P from stromal, endothelial, and cancer cells, it is possible that these transporters may play an important role in the pathological processes regulated by S1P and may worsen the biology of cancer cells. Considering that ABCB1 does not have a role for S1P transport, targeting ABCC1 and/or G2 may be a more promising treatment option for cancer patients. In contrast to the ABC transporters, with some of them originally described as multidrug resistant genes, the roles of Spns2 in cancer remain unknown.

Spns2, a 549 amino acids protein, belongs to the MFS transporter family as determined based on its predicted amino acid sequence [49]. The crystal structure of Spns2 and the precise mechanism of S1P transport via this transporter are still under investigation. S1P import by this transporter also remains to be described. To elucidate the role of Spns2 in cancer, we are currently in the midst of a process of developing gene targeting techniques utilizing nanoparticles to downregulate spns2 expression in cancer cells. How effective this may be in light of other transporters that contribute to S1P export, particularly* in vivo* setting, is yet to be determined. Further investigation is needed to clarify the function and role of Spns2 in normal physiological conditions and in the pathological condition of cancer.

The ABC transporter family and Spns2 are found in various types of cells and have different roles in each type of cell, so that it is possible that targeting a specific transporter may present some difficulties for cancer therapy for patients. In addition to targeting S1P transporters, a possible strategy for cancer treatment involves targeting S1P itself using a monoclonal antibody. In an animal model, neutralization of systemic S1P using Sphingomab, an anti-S1P monoclonal antibody, was shown to suppress lung metastasis [70]. The humanized version of Sphingomab, Sonepcizumab/ASONEP [71], has finished phase I trials and has recently entered phase II efficacy and safety studies for the treatment of renal cell carcinoma and age-related macular degeneration [72]. Targeting S1P itself may be one of the promising strategies for controlling cancer progression and metastasis.

## 5. Conclusion

S1P promotes a variety of intracellular and extracellular biological functions and is transported from inside cells through several members of the ABC transporter family and the enigmatic Spns2 transporter. Once through the plasma membrane, S1P exerts its “inside-out” signaling in the context of the TME and promotes cancer progression. Targeting S1P transporters and S1P signaling in cancer progression is a promising direction for development of the next generation of cancer therapeutics.
